# Supplementary material for: Two-dimensional tessellation by molecular tiles constructed from halogen–halogen and halogen–metal networks
Source: Nat Commun. 2018 Nov 19;9:4871. doi: 10.1038/s41467-018-07323-6 (PMC6242836; doi:10.1038/s41467-018-07323-6)
Supplement: Supplementary file 1 — Supplementary Information [file 41467_2018_7323_MOESM1_ESM.pdf]

Supplementary Information

Two-Dimensional Tessellation by Molecular Tiles Constructed from Halogen-Halogen and Halogen-Metal Networks

Cheng et al.

Supplementary Figures

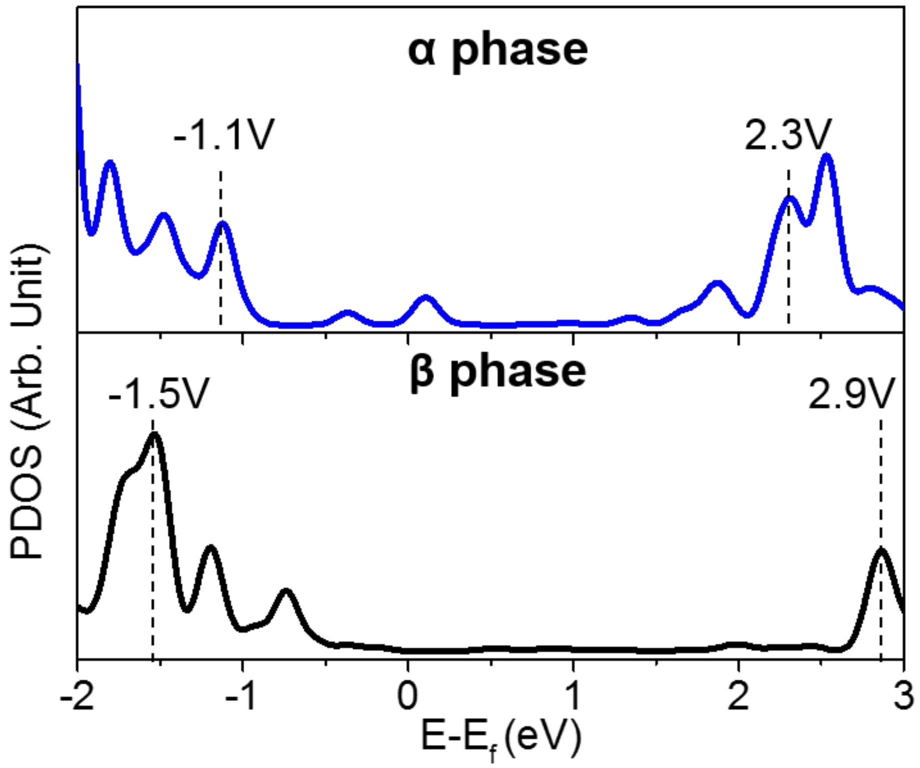

Supplementary Figure 1. Calculated frontier orbitals of I atoms for α and β phases. Upper panel: α phase; lower panel: β phase. The Fermi level of Au substrate was used as reference. The highest occupied state (HOMO) and the lowest unoccupied (LUMO) states of I-atoms are located at -1.1V and 2.3V in α phase, while they are at -1.5V and 2.9V in β phase.

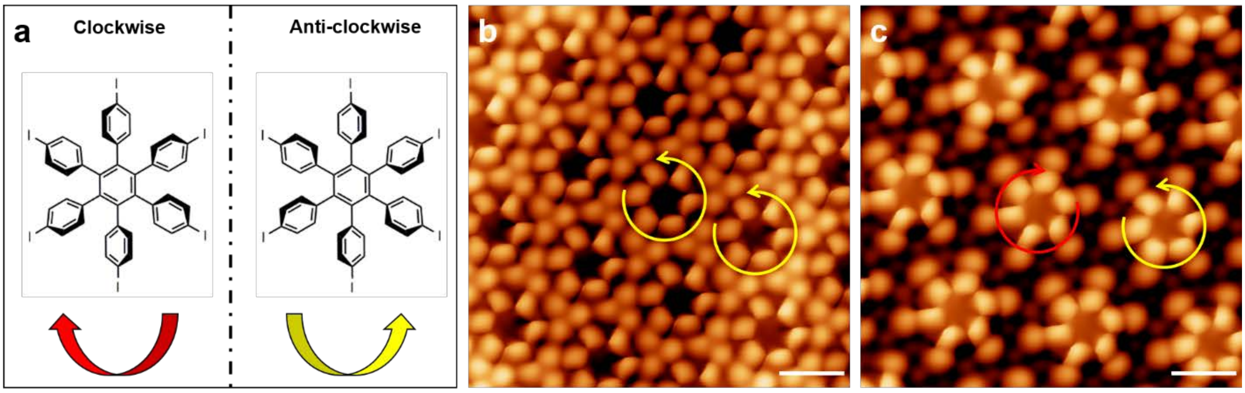

Supplementary Figure 2. Chirality of single HPBI molecule. a, The chemical structure representing chirality of single HPBI molecule upon adsorption on surface. The six out-of-plane phenyl groups adopt clockwise or anti-clockwise rotation with respect to the axis perpendicular to the surface plane. **b,** High-resolution STM image of α phase. The chirality of single molecule can be distinguished by the rotation of six phenyl groups. All the molecules present anti-clockwise orientation (yellow arrow) in α phase. **c,** High-resolution STM image of β phase. Both clockwise (red arrow) and anti-clockwise (yellow arrow) molecules can be found in β phase. Scale bar in all STM images: 1nm.

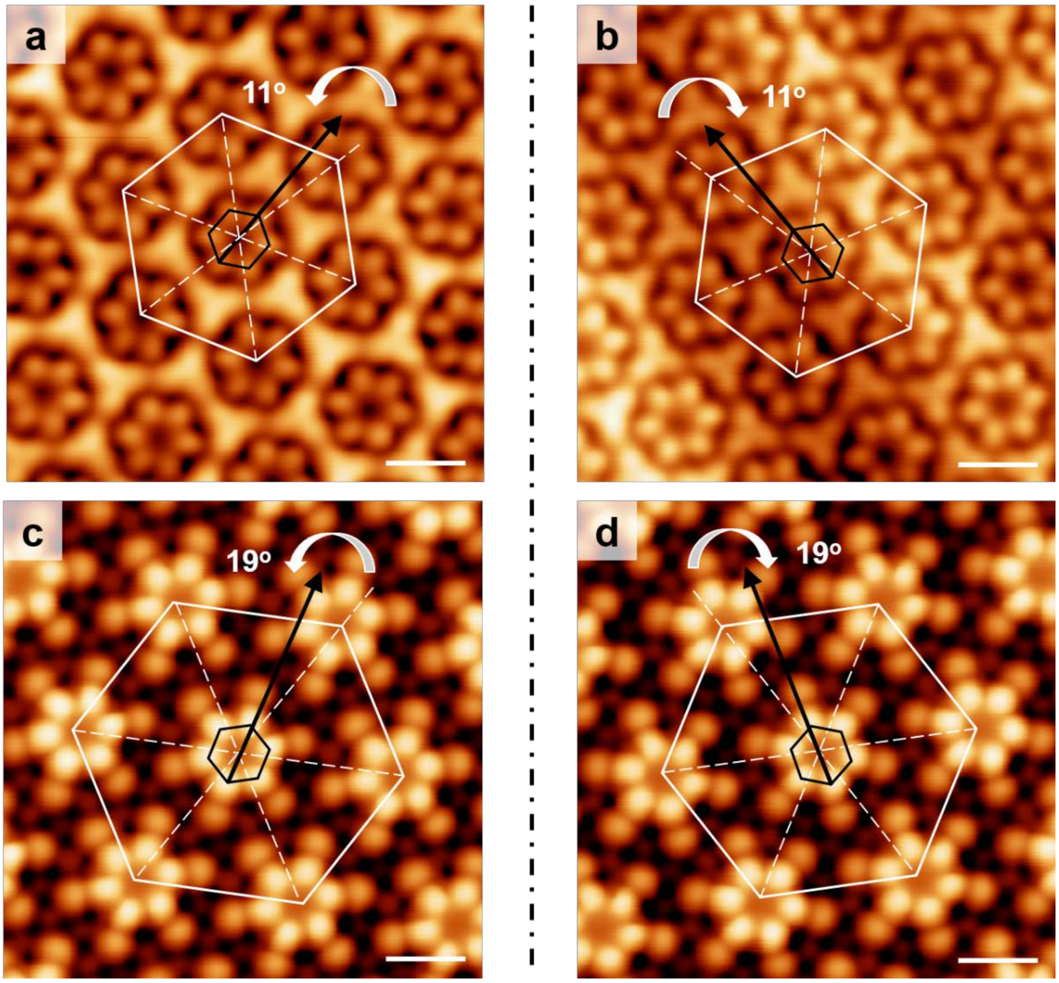

Supplementary Figure 3. Chirality of self-assembly α and β phases. **a, b,** STM images of two enantiomorphous domains of α phase. The black and white hexagons mark the orientation of the single molecule and the self-assembly patterns, respectively. The black arrows and the elongated dashed white lines highlight one of the diagonals in the black and white hexagons. The relative rotation of 11° between these two types of hexagons define the chirality of α phase. **c, d,** STM images of two chiral molecular arrangements of β phase. The relative rotation between the black and white hexagons is 19°. The chirality of α and β phases originate from the chiral arrangements of the molecular building blocks. Scale bar in all STM images: 1nm.

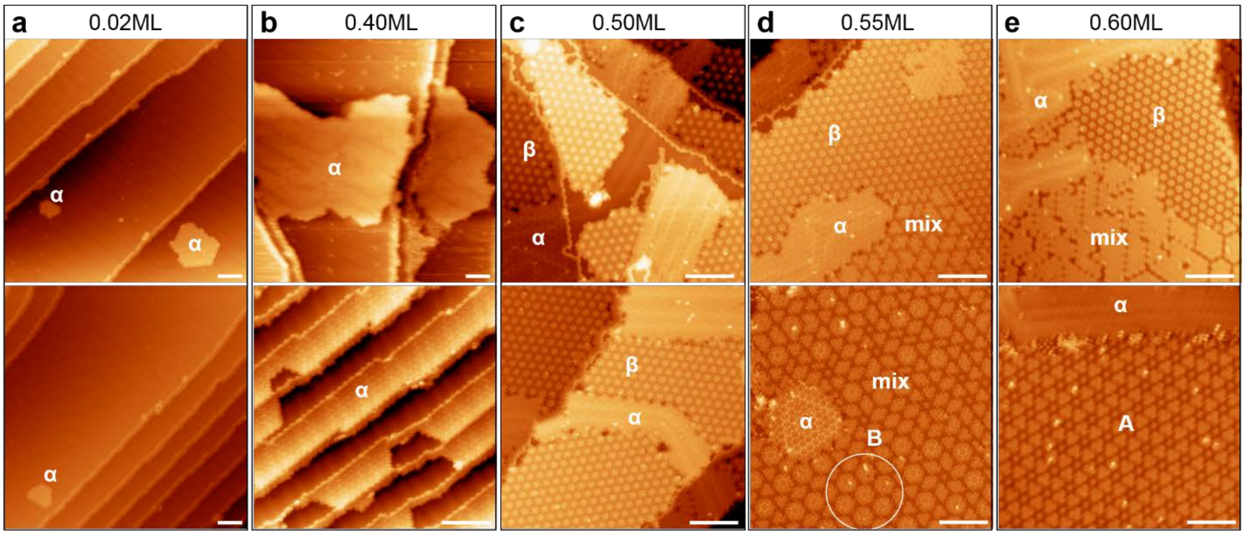

Supplementary Figure 4. Phase evolution of HPBI on Au(111) under different coverages. a, b, c, d, e, STM image of Au(111) after deposition of HPBI at 0.02ML (a), 0.40ML (b), 0.50ML (c), 0.55ML (d) and 0.60ML (e). The different phases are labelled in images. Scale bar: 10nm. At low coverage, only α phase is observed. When increasing the coverage to about 0.50ML, β phase emerges and coexists with α phase. Further increasing the coverage, some disorder mix phases, and the ordered structure A, B, C appear on surface.

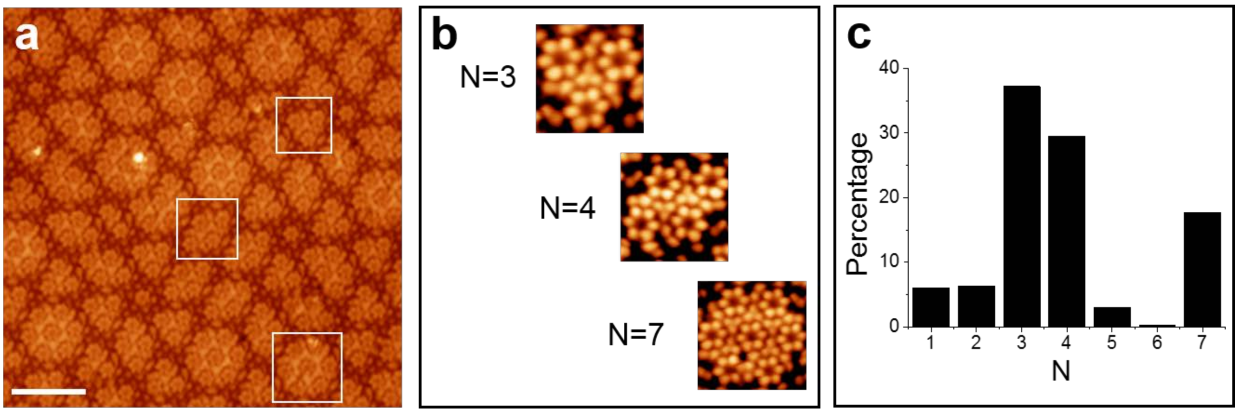

Supplementary Figure 5. Non-periodic mixture of α and β phases. a, STM image of non-periodic mixture of α and β phases, exhibiting molecular clusters containing different numbers of molecule. Scale bar: 5nm. **b,** High resolution STM images of three representative molecular clusters: triangular-shaped trimer, parallelogram-shaped tetramer, and hexagonal-shaped heptamer. N denotes the number of molecule in the cluster. **c,** Statistical analysis of different surface areas with the non-periodic mixed-phase structures. The molecular clusters with N = 3, 4, 7 (corresponding to STM images in (b)) show the highest probabilities in the non-periodic mixed-phase structures.

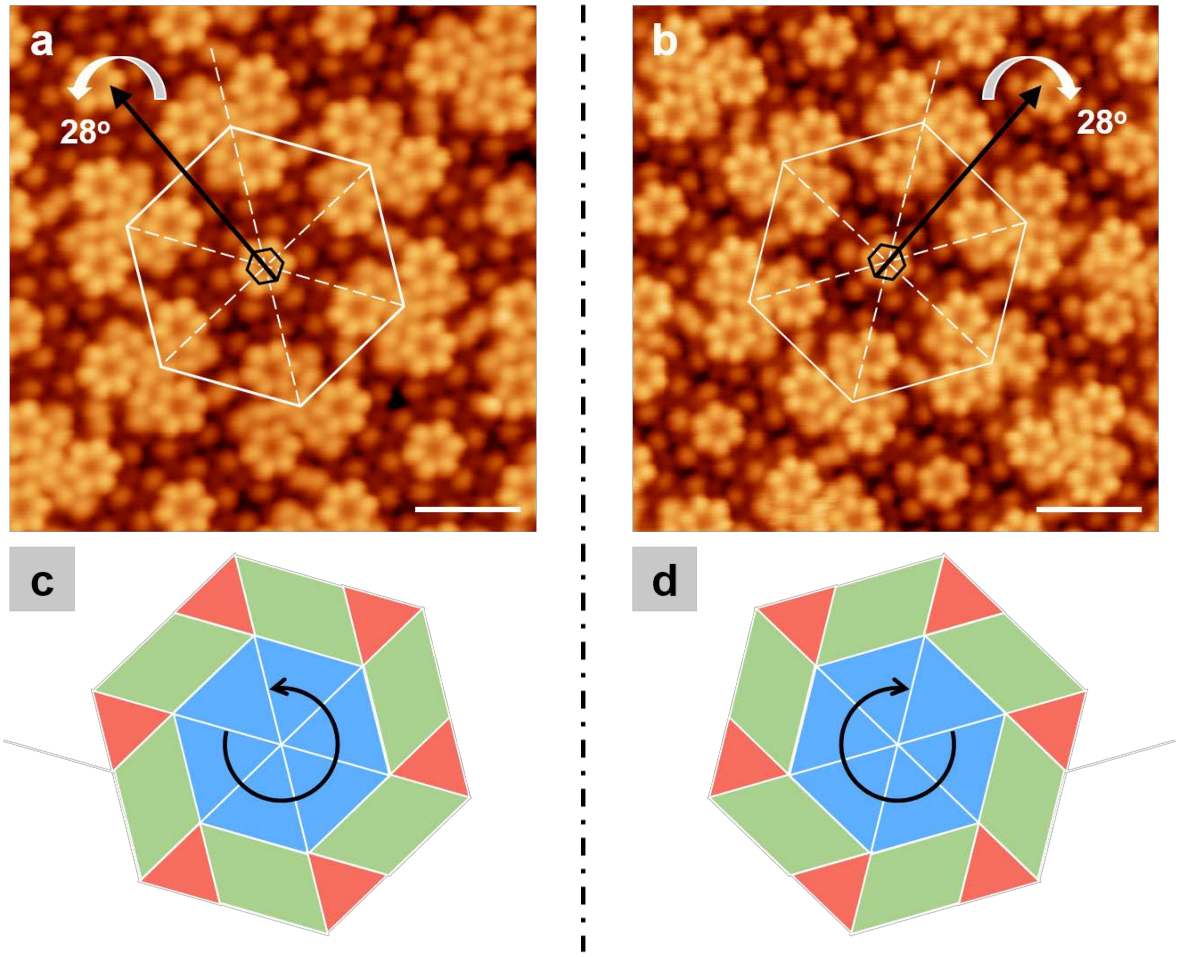

Supplementary Figure 6. Enantiomorphous domains of structure A. a, b, STM images of two enantiomorphous domains of structure A. The chirality of structure A can be expressed by 28° rotation between single molecule (black hexagon) and supramolecular arrangements (white hexagon). **c, d,** The corresponding tessellation representations of (a) and (b). The chirality of structure A can also be recognized by the rotation of the triangular subunits (red triangles) with respect to the hexagon (blue hexagon) symmetry axes. Scale bar in all STM images: 2nm.

Supplementary Method

All reagents and starting materials were obtained from commercial suppliers and used without further purification. Anhydrous dichloromethane (DCM) was distilled from CaH_2. The ^{1}H NMR and ^{13}C NMR spectra were recorded in solution of CD_2Cl_2 on Bruker DPX400 NMR spectrometer. The following abbreviations were used to explain the multiplicities: s = singlet, d = doublet, t = triplet, m = multiplet.

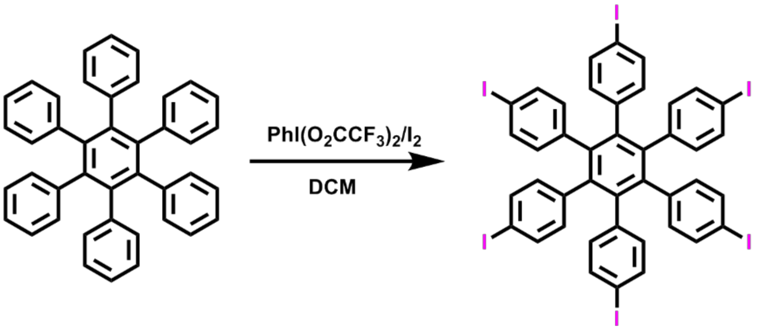

Compound **HPBI** were prepared according to the literature[1]. A mixture of hexaphenylbenzene (3.00 g, 5.61 mmol), $PhI(O_2CCF_3)_2$ (7.96 g, 18.51 mmol), and I_2 (4.70 g, 18.51 mmol) in dry DCM (250 mL) under nitrogen atmosphere was stirred at room temperature overnight in the dark, and then hexane was added to the reaction mixture. The resulting precipitate was filtered and washed with hexane. This solid was dissolved in $CHCl_3$, which was washed with aqueous $Na_2S_2O_4$ and brine and dried over Na_2SO_4. After evaporation of solvent, the solid residue was recrystallized from $CHCl_3$-hexane to give white solid **HPBI** (6.15 g, 85%).

^{1}H NMR (400 MHz, CD_2Cl_2, δ ppm): 7.25 (d, 12H, *J* = 8.4 Hz), 6.52 (d, 12H, *J* = 8.4 Hz).

^{13}C NMR (100 MHz, CD_2Cl_2, δ ppm): 139.40, 139.25, 136.17, 132.91, 91.65.

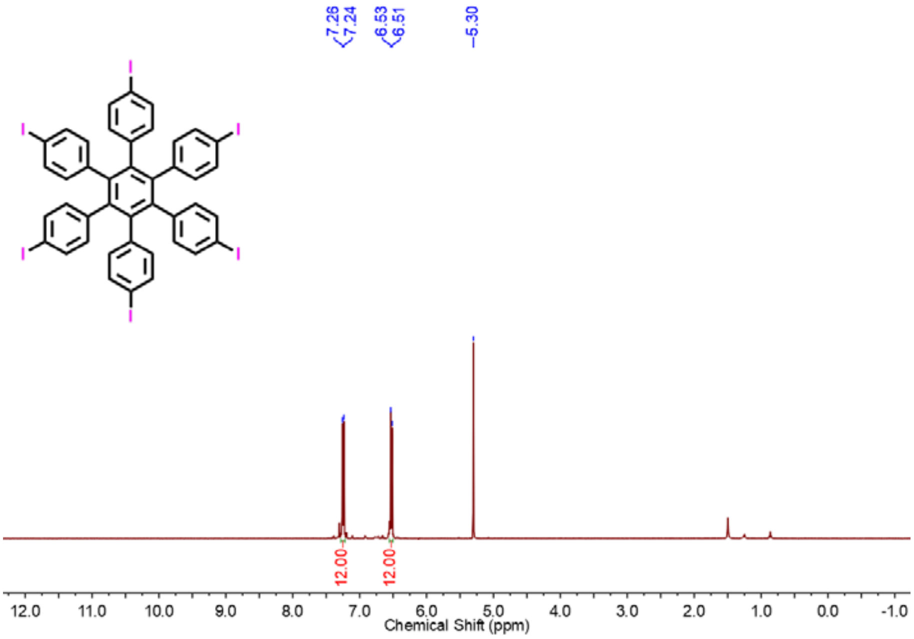

^{1}H NMR spectrum (400 MHz) of HPBI in CD_2Cl_2 at 298 K.

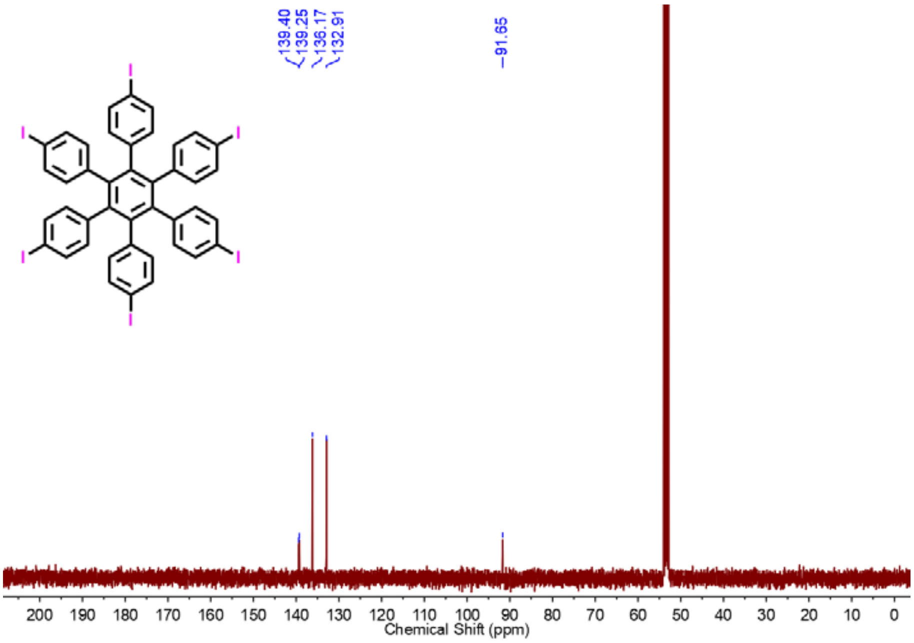

^{13}C NMR spectrum (400 MHz) of HPBI in CD_2Cl_2 at 298 K.

Single crystal of HPBI was grown through slow diffusion of ethanol into its $CHCl_3$ solution under ambient condition. A colorless single crystal with the dimension of (0.103 × 0.198 × 0.235) mm^3 was used for the X-ray crystallographic analysis. The data was collected with

monochromatized Mo-Kα radiation (0.71073 Å) at 100 K, using a four circles goniometer Kappa geometry, Bruker AXS D8 Venture, equipped with a Photon 100 CMOS active pixel sensor detector. The frames were integrated with the Bruker SAINT software using a narrow-frame algorithm. The structure was solved in the orthorhombic unit cell and refined using the SHELXT, VERSION 2014/5 Software[2]. Refinement was carried out by least squares procedures on weighted F2 values using the SHELXL-2014/7 (Sheldrick, 2014)[3] included in the APEX3 v2016, 9.0, AXS Bruker program. Hydrogen atoms were localized on difference Fourier maps but then introduced in the refinement as fixed contributors in idealized geometry with an isotropic thermal parameters fixed at 20 % higher than those carbons atoms they were connected. The results of the refinement and selected bond lengths of these molecules are listed in Tables 1-2. Crystallographic data for HPBI is deposited in the Cambridge Crystallographic Data Center with number CCDC-1843494.

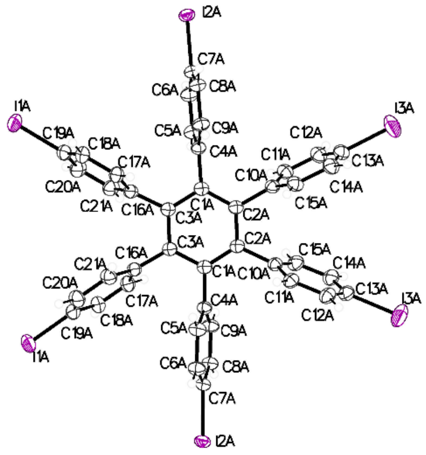

X-ray crystal structure of HPBI.

Supplementary Table 1. Crystal data and structure refinement for HPBI.

**Identification code**	HPBI
**Chemical formula**	$C_{42}H_{24}I_6$
**Formula weight**	1290.01 g/mol
**Temperature**	100(2) K
**Wavelength**	0.71073 Å
**Crystal size**	(0.103 x 0.198 x 0.235) mm^3
**Crystal system**	orthorhombic
**Space group**	I b c a

**Unit cell dimensions**	a = 16.423(3) Å	α = 90°
	b = 17.330(3) Å	β = 90°
	c = 27.344(3) Å	γ = 90°
**Volume**	7782.(2) $Å^3$	
**Z**	8	
**Density (calculated)**	2.202 g/cm^3	
**Absorption coefficient**	4.819 mm^{-1}	
**F(000)**	4752	
**Theta range for data collection**	2.27 to 28.28°	
**Index ranges**	-21<=h<=20, -23<=k<=21, -33<=l<=36	
**Reflections collected**	35119	
**Independent reflections**	4838 [R(int) = 0.0402]	
**Coverage of independent reflections**	99.9%	
**Absorption correction**	Multi-Scan	
**Max. and min. transmission**	0.6370 and 0.3970	
**Refinement method**	Full-matrix least-squares on F^2	
**Refinement program**	SHELXL-2014/7 (Sheldrick, 2014)	
**Function minimized**	Σ w(F_o^2 - F_c^2)^2	
**Data / restraints / parameters**	4838 / 1022 / 435	
**Goodness-of-fit on F^2**	1.069	
**Final R indices**	3602 data; I>2σ(I)	R1 = 0.0418, wR2 = 0.1002
	all data	R1 = 0.0634, wR2 = 0.1120
**Weighting scheme**	w=1/[$\sigma^2(F_o^2)+(0.0406P)^2+97.4012P$] where P=($F_o^2+2F_c^2$)/3	
**Largest diff. peak and hole**	1.458 and -1.439 $eÅ^{-3}$	
**R.M.S. deviation from mean**	0.139 $eÅ^{-3}$	

Supplementary Table 2. Bond lengths (Å) for HPBI.

C1A-C2A	1.397(7)	C1A-C3A	1.409(7)
C1A-C4A	1.472(7)	C2A-C2A	1.400(12)
C2A-C10A	1.507(7)	C3A-C3A	1.433(11)
C3A-C16A	1.488(7)	I1A-C19A	2.103(5)

C16A-C17A	1.383(5)	C16A-C21A	1.388(5)
C17A-C18A	1.386(5)	C17A-H17A	0.93
C18A-C19A	1.378(5)	C18A-H18A	0.93
C19A-C20A	1.365(5)	C20A-C21A	1.405(5)
C20A-H20A	0.93	C21A-H21A	0.93
I2A-C7A	2.108(4)	C4A-C5A	1.384(5)
C4A-C9A	1.387(5)	C5A-C6A	1.387(5)
C5A-H5A	0.93	C6A-C7A	1.377(5)
C6A-H6A	0.93	C7A-C8A	1.368(5)
C8A-C9A	1.400(5)	C8A-H8A	0.93
C9A-H9A	0.93	I3A-C13A	2.104(5)
C10A-C11A	1.383(5)	C10A-C15A	1.388(5)
C11A-C12A	1.387(5)	C11A-H11A	0.93
C12A-C13A	1.378(5)	C12A-H12A	0.93
C13A-C14A	1.365(5)	C14A-C15A	1.406(5)
C14A-H14A	0.93	C15A-H15A	0.93

Supplementary References

1. Kobayashi, K. *et al.* Syntheses of Hexakis(4-functionalized-phenyl)benzenes and Hexakis[4-(4'-functionalized- phenylethynyl)phenyl]benzenes Directed to Host Molecules for Guest-Inclusion Networks. *J. Org. Chem.* **70**, 749-752 (2005).
2. Sheldrick, G. SHELXT - Integrated space-group and crystal-structure determination. *Acta Cryst.* **A71**, 3-8 (2015).
3. Sheldrick, G. Crystal structure refinement with SHELXL. *Acta Cryst.* **C71**, 3-8 (2015).
